# Description and Characterization of Three-Dimensional Human Mast Cell Progenitor Spheroids In Vitro

**DOI:** 10.7759/cureus.53708

**Published:** 2024-02-06

**Authors:** Rebecca Praetzel, Mona Motaghed, Mohammad Fereydouni, Elnaz Ahani, Chris Kepley

**Affiliations:** 1 Department of Molecular and Cellular Sciences, Liberty University College of Osteopathic Medicine, Lynchburg, USA; 2 Department of Nanoengineering, North Carolina Agricultural and Technical State University, Greensboro, USA; 3 Department of Nanoscience, University of North Carolina at Greensboro, Greensboro, USA

**Keywords:** culturing protocol, spheroids, progenitor, adipose tissue, human mast cell

## Abstract

Human mast cells (MC) are an essential component of the immune system as they uniquely store and release a wide range of soluble mediators through IgE and non-IgE mechanisms. Several tissue sources can be used to differentiate functional MC for *in vitro* and *in vivo* studies. Here we describe an improved method for obtaining large numbers of human MC from adipose tissue with advantages over current methods. We analyzed donor parameters (e.g. age, race) on MC-isolation following adipose and skin tissue digestion from healthy donors. Adipose and skin-derived MC were morphologically and immunophenotypically similar in all donors regardless of age. However, donor-dependent variations in MC numbers were observed following tissue digestion. In addition, we identified and characterized three-dimensional structures from which mature MC emerged *in vitro* using peripheral blood and human tissue sources. MC progenitor spheroids (MCPS) appeared approximately one week following progenitor isolation and were consistently observed to have mature MC attached, emerging, or nearby when cultured in a stem cell factor-containing medium. The overall characteristics of the MCPS were similar from each tissue source. We propose that these MCPS serve as the common source of human MC *in vitro*.

## Introduction

Mast cells (MC) are involved in innate and acquired immunity and are located in virtually all organs close to host-environment interfaces, such as vascular and mucosal barriers, nerve endings, and skin [1]. They are most recognized as mediators of Type I hypersensitivity as they (along with basophils) uniquely store and release histamine following FcεRI and non-FcεRI challenges [2,3]. Mast cells also pre-store TNF-α and generate newly-formed immune mediators such as cytokines, leukotrienes, and prostaglandins and are thus uniquely poised in most tissues to mediate innate and adaptive immune responses. Many studies have described the *in vitro* differentiation of primary human MC from progenitor cells isolated from bone marrow [4-6], skin [7,8], lung [9,10], heart [11,12], intestine [13,14], umbilical cord blood and venous blood [15-17], fetal liver [18,19], and uterus [15]. Adipose tissue from liposuction surgery is another source that can be obtained for potential autologous purposes [20-22], such as adoptive cell transfer for cancer immunotherapy [23-25]. These methods are labor-intensive, have unintended infections, and have cell numbers and yield variations, which appear to be donor-dependent [22,26].

Here, we describe an improved method to isolate MC from human adipose tissue that is less time-consuming and not reliant on the conditioned medium described previously [20] or the cytokines IL-3 or IL-6 used in many protocols. In addition, we highlight clear age-dependent differences between donors in MC numbers obtained in culture. Moreover, three-dimensional structures termed MC progenitor spheroids (MCPS) were identified and characterized in cultures of human adipose, skin, blood, and lung, from which mature MC emerged in the *in vitro* cultures.

## Materials and methods

Tissue digestion and MC progenitor isolation

Human adipose, skin, and lung tissue were processed under IRB approval from the Cooperative Human Tissue Network and approved by the Liberty University Institutional Review Board (IRB-FY21-22-844). Mast cells were dispersed from human lung tissue [27] and skin as described [28]. Lung MC were cultured at 0.5 to 2×10^5^ cells/mL in 24-well plates using RPMI medium with 10% heat-inactivated fetal bovine serum (HI-FCS; Sigma, USA), 10 mmol HEPES, 50 μmol 2-mercaptoethanol, 4 mmol L-glutamine, 100 U/mL penicillin, 100 μg/mL streptomycin, and 80 ng/mL human recombinant stem cell factor (SCF) protein (aa 1-189, His Tag) (SinoBiological, USA). Skin MC were cultured the same except with X-VIVO15 (Lonza, Switzerland) or Aim-V (Gibco, Invitrogen, USA) media with 80 ng/mL SCF. Adipose tissue was processed similarly to previously described protocols with the following differences [20]. Tissue was weighed and placed in a sterile petri dish with 5 mL wash buffer (10% Hanks’ balanced salt solution, 0.04% sodium bicarbonate, 1% HEPES, 1% HI-FBS, 1% Antibiotic-Antimycotic Solution, and 0.5% Amphotericin B. Tissue was minced into small pieces (0.25 cm^3^), placed into 50 mL conical tubes, and 20 mL digestion buffer [20] was added and the tissue was placed on a rotating table at 37°C for two hours. The digested tissue was strained through an eight-inch strainer sieve, washed, and filtered digest placed in 50 mL Falcon tubes and centrifuged for 10 minutes at 360 g and 18°C. The lipid layer and digestion buffer were aspirated to leave 10 mL of buffer at the bottom of the tubes along with pelleted cells and repeat the above process for each tube. The washed pooled pellets were all isolated into one 50 mL tube and filtered through a 100-micron cell strainer to remove the large, undigested pieces of tissue. Red blood cell (RBC) lysis buffer (BioLegend, USA) or Histopaque was used to remove RBCs and washed after tissue digestion, and the cell suspension was placed directly into X-VIVO15 (or Aim-V) with SCF in 24-well plates. In some experiments, human IL-6 (50 ng/mL) and IL-3 (10 ng/mL) were added to the digested cell suspension for three weeks and replaced with SCF-medium only. Half the media was replaced approximately every ten days for all cultures, and cells split when MC are 50-75% confluent on the plate bottom. Toluidine blue staining of cytocentrifuged cells on cytospin slides was used to assess MC maturation as described previously [20]. In some experiments, human peripheral blood-derived MC (BDMC) were obtained from healthy adult volunteers as described after informed consent was obtained under protocols approved by the Institutional Review Board of the National Institute of Allergy and Infectious Diseases (protocols 2009-I-0049 and 10-I-0196) [29] and provided by Dr. Dean Metcalfe.

mRNA detection

Reverse transcription polymerase chain reaction (RT-PCR) was performed using the Qiagen OneStep RT-PCR kit (Qiagen, Germany) previously described [30]. All primers were purchased from Integrated DNA Technologies (IDT, USA). Cycling conditions were 50°C for 30 minutes, 95°C for 15 minutes, followed by 35 cycles of 94°C for 45 s, 53-63°C for 45 s (according to primer Tm), 72°C for 1 minute, and a final 10 minutes extension at 72°C, as described previously [20].

MCPS immunohistochemistry (IHC)

Immunohistochemistry was performed as described previously [31,32]. Briefly, cytocentrifuged cells were fixed in methanol for 10 minutes at -20°C and endogenous peroxidase was blocked by incubation with 0.6% H_2_O_2_ in methanol for 30 minutes at room temperature, washed in PBS, (pH 7.4), and normal goat serum (1:100 in PBS/0.5% BSA) added for one hr. Slides were washed and incubated overnight in a humid chamber at 4°C with primary mouse antibodies (Abs) to human tryptase, chymase, CD34, or isotype control MOPC (1 μg/mL; Sigma-Aldrich). After washing in PBS, slides were incubated with HRP-conjugated goat anti-mouse IgG, and peroxidase was visualized using 3-amino-9-ethyl carbazole as described [32]. Positively stained cells developed a reddish-brown color. The color threshold analysis was measured using Image J software.

Scanning electron microscopy (SEM)

Cells were fixed on glass coverslips, washed with 70% ethanol, and air-dried at room temperature. Samples were fixed with 25% glutaraldehyde and 40% formaldehyde in PBS for two hours, dehydrated in a gradient series of ethanol (50% to 100%), and further dehydrated using a critical point dryer. Specimens were mounted on stubs using conductive double-sided carbon tape and coated with 10 nm thick gold-palladium by a sputter coater (Leica Microsystems, IL, USA). Cells were examined using a field emission scanning electron microscope (Zeiss Auriga FIBFESEM, Zeiss, NY, USA) at 4 kV.

Time-lapse imaging of MC emerging from MCPS

Confocal imaging was used to investigate the MC originating from MCPS in real time. MCPS were incubated with a culture medium containing 500 nM CellTracker Deep Red (ThermoFisher Scientific) for 30 minutes. MC were washed, resuspended in warm X-VIVO15 plus SCF (80 ng/mL), and placed in a live cell incubator affixed to a Zeiss AXIO Observer Z1 Spinning Disc Confocal Microscopy. A representative adipose progenitor was selected, and images were taken every 10 minutes over 24 hrs.

Functional characterization of adipose MC

The degranulation of MC was measured as described [20]. Briefly, MC activated 2 μg/mL anti-FcεRI antibody (3G6) for 30 minutes (degranulation) and β-hexosaminidase release was measured [33].

Statistical analysis

All experiments were performed in triplicate from three separate donors. One-way ANOVA was used to compare three or more groups within one variable. To analyze two groups, the unpaired t-test was used. The data are expressed as mean ± standard deviation. P-values < 0.05 were considered statistically significant. Experimental data were analyzed using GraphPad Prism software (GraphPad Software, San Diego, CA).

## Results

**Figure 1:**
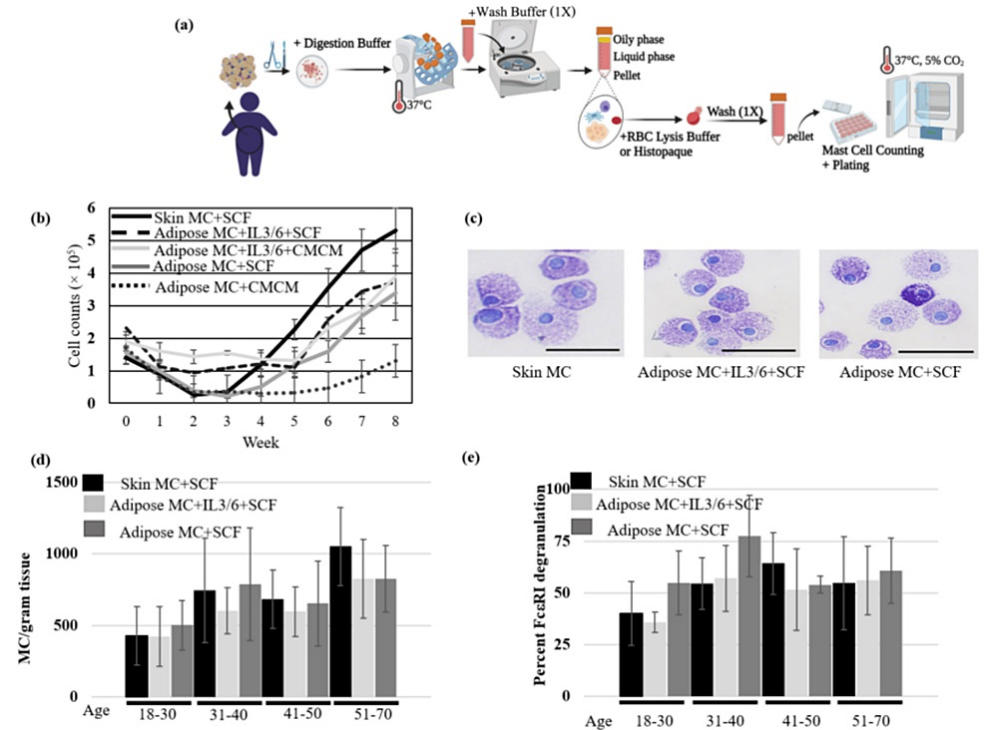
Donor- and time-dependency in primary human MC isolation from tissue. (a) Workflow diagram of human adipose-derived MC isolation. (b) MC proliferation over time. 30 g of the skin or adipose tissue from 18-30-year-old donors were digested and cells were seeded in the 24-well plates (2.5x10^5^ cells per well) under the indicated conditions. MC from all conditions were counted every week for eight weeks (n=3). (c) Toluidine blue staining shows mature MC isolated from skin or adipose tissue (bar size: 20 μm). (d) Relationship between the number of isolated MC and the age of donors. 30 g of the skin or adipose tissue from 18-70-year-old donors were digested and seeded in the 24-well plates (2.5×10^5^ cells per well) under the indicated conditions. MC were counted every week for eight weeks (n=3). (e) The effect of donor age at the β-hexosaminidase release of adipose (n=10) and skin (n=10) MC. Adipose or skin MC (10^5^ cells per condition) were challenged with 2 μg/mL anti-FcεRI Abs and the degranulation assessed in the supernatants. Statistical data analysis showed no difference was observed at adipose- and skin-derived MC degranulation in each donor age group (P-value < 0.5) Error bars represent ± SD. MC: Mast cells; SCF: stem cell factor

Previously, it was demonstrated that functional MC could be obtained from adipose tissue and used to trigger cancer cell apoptosis through FcεRI [20]. However, initial protocols required the use of the conditioned MC medium to induce MC from adipose tissue. In addition, the differentiation of MC from other tissue sources utilized IL-6 and IL-3 early in the culture process [21]. We hypothesized that adipose-derived MC differentiation could be induced without a need for a conditioned medium or additional cytokines besides SCF. In this improved protocol, the approximate time between tissue acquisition and progenitor isolation was reduced, the requirement for non-SCF cytokines was eliminated, and no conditioned medium from MC cultures was required [34] (Figure 1a). As seen in Figure 1b, there was no significant (P<0.05) difference in culturing adipose tissue directly into SCF-containing medium compared to conditioned medium or IL-3/IL-6-cultured progenitor cells after eight weeks. The early decline in MC may be due to the formation of MCPS that attach to the MC. Morphology as assessed using metachromatic dyes revealed no obvious differences in phenotype (Figure 1c). The number of MC obtained from tissue (Figure 1d) and the degranulatory response to the FcεRI challenge (Figure 1e) was similar among each age group with no statistically significant differences observed.

MCPS: a common structure from which MC emerge from *in vitro*

**Figure 2:**
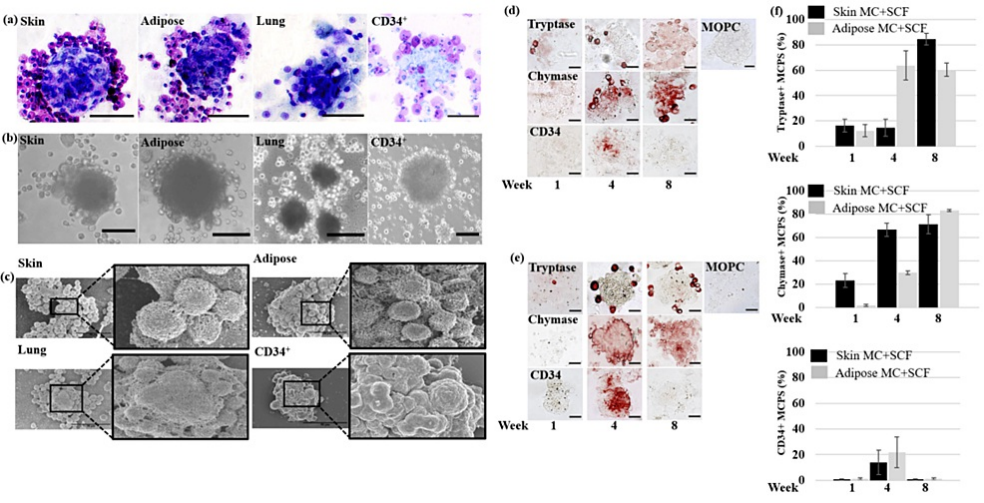
Morphological and phenotypic characterization of MCPS. Samples from MC cultures derived from the indicated tissue at four weeks were stained with (a) Toluidine blue (bar size: 50 μm) or visualized using (b) phase-contrast (bar size: 50 μm) or (c) scanning electron microscopy. Experiments representative of three donors.  Immunohistochemistry staining in (d) skin and (e) adipose MC and MCPSs vs. cell culture time showed a positive trend in tryptase and chymase development in adipose and skin MC and MCPS. However, the CD34 marker decreased in the early ages of MCPS development and MC production. MOPC was used as a negative control in all experiments. (f).  Tryptase, chymase, and CD34 marker development graphs vs. time-based on the IHC study. All data analysis has been conducted with Image-J and GraphPad Prism software (n=3). Scale bar: 50 μm. n denotes the number of donors. Error bars represent ± SD. MC: Mast cells; MCPS: mast cell progenitor spheroids; SCF: stem cell factor

Large bundles of cells forming three-dimensional spheroids from which the mature MC emerged were observed in each of the cultures. These MCPS are a common structure that appear from the skin, adipose, lung, and blood approximately one week following tissue digestion or isolation from blood. Figure 2a illustrates the metachromatic staining while Figure 2b represents the phase contrast images of the skin, adipose, lung, and blood MC and MCPS. As shown in Figure 2c, higher magnifications using SEM revealed high numbers of rounded MCPS from each tissue source and mature MC attached. The MCPS had similar average diameter size of adipose (120 ±79 u​​m) and skin MCPS (114 ±41 um) (not shown). The average number of MCPS per gram of tissue four weeks after cell culture from skin and adipose was 650±24 um and 415±40 um, respectively. Large numbers of MC can be observed on and around the MCPS, with new mature MC continually emerging off random areas of the MCPS four weeks after cell culture (Supplemental Video 1). The time course for the appearance of representative MC-specific surface markers and the decline in stem-cell markers in the MCPS was investigated. The MC-specific marker tryptase was detectable one week following tissue digestion of both skin (Figure 2d) and adipose (Figure 2e), confirming the presence of mature MC in these tissues described previously [20, 32]. By eight weeks, both adipose and skin MCPS were >60% positive for tryptase (Figure 2f; top) and >70% positive for chymase (Figure 2f; middle). The MCPS transiently expressed CD34 starting at week two, which decreases to no staining observed at eight weeks (Figure 2f; bottom). Lung MCPS had a similar IHC expression (data not shown). Thus, the MCPS appears to be a common structure from which mature MC emerge in culture from various tissue sources. 

## Discussion

An improved protocol to obtain human MC was described. This new protocol produced phenotypically, morphologically, and functionally mature MC and was reproducible, time-saving, and more economical than other methods described previously [20,22,35,36]. A common 3D structure from which mature MC emerged *in vitro* was identified and characterized. The perpetuation of these cultures appeared to depend on the formation of MCPS.

It was initially hypothesized that MC were derived from an undifferentiated mesenchymal stem cell [37]. In 1977, Kitamura's group revealed that MC were from a hematopoietic stem cell origin [38], and mature MC arise from a common progenitor depending on their anatomical location [39]. Human MC arise from bone marrow as CD34+ [4,40] and CD34+/CD117+ [21,41,42] progenitor cells at an immature stage in a process highly regulated by transcription factors [39,40]. The MC progenitors enter the circulation as granular mononuclear leukocytes and are recruited into peripheral tissues with chemokines secreted by tissue stromal cells [43].

The emergence of MCPS in SCF-containing medium appears to be unique to *in vitro* cultures. No MCPS have been described* in vivo* that we are aware of. We did not find evidence these exist in non-pathological or pathological (e.g. mastocytosis) conditions. There are studies suggesting large clumps of mature MC or increased numbers of MC are present in bone marrow from patients with mastocytosis but do not appear to be MCPS [44-46]. Thus, the presence of MCPS may be limited to *in vitro* cultures in which connective tissue and other matrices are not present allowing for the formation of the structures. 

Studies examining MC development, phenotype, and functional responses in mice differ considerably compared to human MC [15,47,48]. For example, mouse MC have a diverse range of proteases [47,49], while human MC express fewer (e.g. tryptase, chymase, and carboxypeptidase-A) [21,50,51]. The expression and functional response of MC Fc receptor expression are different, and mouse FcεRI does not bind to human IgE marrow from mastocytosis patients and has been observed to have large clumps of mature MC or increased numbers of MC [8,52,53]. Mouse MC have a different expression profile for Ig receptors than human MC. They can be activated by FcεRI and FcγRIII while human MC are only activated by FcεRI unless manipulated by cytokines [54]. Histamine is released from human MC, while serotonin and histamine are liberated from these cells in mice [55]. Interleukin-3 profoundly affects murine MC differentiation and function not observed with human MC [56,57]. There appear to be alternative progenitor sources in mice from which cells can give rise to MC in tissues such as the skin, white adipose tissue, and intestine [44,58]. In humans, MC arises from CD34+CD117+ progenitor cells. Shimizu et al. [5] isolated CD34+CD117- progenitor cells (from humans) cultured with SCF and IL-6 resulted in monocytes/macrophage development, while CD34+CD117+ progenitor cells gave rise to human MC. Primary mouse MC cultures did not appear to have MCPSs from which MC emerged [59,60], adding another difference between the two systems [47,61]. Many human MC lines have been identified, including HMC-1 (human mast cell leukemia-1) [62], LAD2 (Laboratory of Allergic Diseases 2) [63], LUVA (Laboratory of University of Virginia) [64], ROSA [65], and LADR [66]. These cell lines exhibit certain specific similarities to primary human MC, but inconsistencies related to variable FcεRI densities, functional responses to FcεRI challenge, granule formation, growth rates, and phenotypic drift offer challenges in their use [15,26].

There is thus a continuing need for cellular MC models that best predict MC biology in health and disease. In addition, our laboratory is using autologous MC for potential use as a new strategy for cancer immunotherapy [20,23-25]. Recently, we used the above protocol to obtain human MC from adipose tissue and sensitized them with tumor-specific IgE to show they could induce apoptosis of cancer cells in vitro and shrink tumors/increase the lifespan of mice in vivo using patient-derived tumor cells implanted in immunocompromised mice without evidence of toxicity [24]. This new approach using autologous MC as new cancer immunotherapy is premised on efficient, autologous production of MC, tumor targeting (using tumor-specific IgE or chimeric antigen strategies), and controllable MC degranulation leading to tumor-specific killing. The MC cultures can also be cryo-preserved indefinitely without significant loss of viability and genetically manipulated to increase anti-tumor and/or decrease toxic mediators (data not shown), which provides further advantages for research and autologous applications. While cultures from adipose and skin can proliferate for up to 10 months and produce >10^9^ MC, depending on the donor, research to immortalize these cells is underway so as to negate the need for SCF.

These studies are limited in that the isolation of specific MCPS was not performed which prevented more in depth phenotypic and functional studies from being done. In addition, the observation that MCPS only form in vitro limits the translational significance of the findings.

## Conclusions

In conclusion, an improved protocol is described for culturing human MC from tissue that offers several advantages over current methods. MCPS from which mature MC emerge in cultures from various tissue sources suggest a common way in which SCF-containing MC cultures result in homogeneous populations. These results provide further knowledge regarding MC ontogeny and another method for researchers to obtain human MC for studying phenotypic and functional parameters.

## Appendices

Supplemental Material
